# Ethyl 3-[6-(4-meth­oxy­benzene­sulfon­amido)-2*H*-indazol-2-yl]propano­ate monohydrate

**DOI:** 10.1107/S1600536812051975

**Published:** 2013-01-09

**Authors:** Najat Abbassi, El Mostapha Rakib, Abdellah Hannioui, Mohamed Saadi, Lahcen El Ammari

**Affiliations:** a Laboratoire de Chimie Organique et Analytique, Université Sultan Moulay Slimane, Faculté des Sciences et Techniques, Béni-Mellal, BP 523, Morocco; b Laboratoire de Chimie du Solide Appliquée, Faculté des Sciences, Université Mohammed V-Agdal, Avenue Ibn Battouta, BP 1014, Rabat, Morocco

## Abstract

In the title compound, C_19_H_21_N_3_O_5_S·H_2_O, the central indazole system is essentially planar (r.m.s. deviation = 0.012 Å), while both the benzene ring and the mean plane defined by the non-H atoms of the ethyl propionic ester unit (r.m.s. deviation = 0.087 Å) are nearly perpendicular to the indazole plane, as indicated by the dihedral angles of 82.45 (8) and 75.62 (8)°, respectively. Consequently, the mol­ecule adopts a U-shaped geometry. In the crystal, the water mol­ecule, which is linked to the indazole system by a strong O—H⋯N hydrogen bond, is also involved in two additional N—H⋯O and O—H⋯O inter­actions, which link the organic mol­ecules into chains along the *b-*axis direction.

## Related literature

For the pharmacological activity of sulfonamides, see: Gadad *et al.* (2000 ▶); Brzozowski *et al.* (2010 ▶); Drew (2000 ▶); Garaj *et al.* (2005 ▶). For their anti­proliferative activity see: Abbassi *et al.* (2012 ▶); Bouissane *et al.* (2006 ▶).
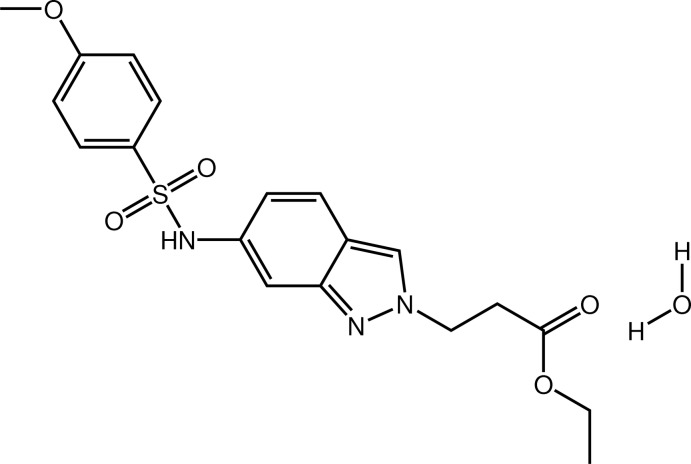


## Experimental

### 

#### Crystal data

C_19_H_21_N_3_O_5_S·H_2_O
*M* *_r_* = 421.46
Monoclinic, 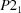

*a* = 9.0248 (3) Å
*b* = 8.7602 (3) Å
*c* = 13.1792 (4) Å
β = 101.062 (2)°
*V* = 1022.58 (6) Å^3^
*Z* = 2
Mo *K*α radiation
μ = 0.20 mm^−1^
*T* = 296 K
0.42 × 0.37 × 0.28 mm

#### Data collection

Bruker X8 APEX diffractometer
10050 measured reflections
4021 independent reflections
3887 reflections with *I* > 2σ(*I*)
*R*_int_ = 0.027

#### Refinement

*R*[*F* ^2^ > 2σ(*F* ^2^)] = 0.031
*wR*(*F* ^2^) = 0.077
*S* = 1.05
4021 reflections
262 parameters
1 restraint
H-atom parameters constrained
Δρ_max_ = 0.28 e Å^−3^
Δρ_min_ = −0.28 e Å^−3^
Absolute structure: Flack (1983 ▶), 1779 Friedel pairs
Flack parameter: 0.03 (6)

### 

Data collection: *APEX2* (Bruker, 2009 ▶); cell refinement: *SAINT* (Bruker, 2009 ▶); data reduction: *SAINT*; program(s) used to solve structure: *SHELXS97* (Sheldrick, 2008 ▶); program(s) used to refine structure: *SHELXL97* (Sheldrick, 2008 ▶); molecular graphics: *ORTEP-3 for Windows* (Farrugia, 2012 ▶); software used to prepare material for publication: *PLATON* (Spek, 2009 ▶) and *publCIF* (Westrip, 2010 ▶).

## Supplementary Material

Crystal structure: contains datablock(s) I, global. DOI: 10.1107/S1600536812051975/lr2095sup1.cif

Structure factors: contains datablock(s) I. DOI: 10.1107/S1600536812051975/lr2095Isup2.hkl

Supplementary material file. DOI: 10.1107/S1600536812051975/lr2095Isup3.cml

Additional supplementary materials:  crystallographic information; 3D view; checkCIF report

## Figures and Tables

**Table 1:** Hydrogen-bond geometry (Å, °)

*D*—H⋯*A*	*D*—H	H⋯*A*	*D*⋯*A*	*D*—H⋯*A*
O6—H6*A*⋯N2	0.86	1.94	2.8029 (19)	176
N1—H1⋯O6^i^	0.81	1.95	2.7575 (19)	177
O6—H6*B*⋯O1^ii^	0.86	2.10	2.9094 (17)	156

Symmetry codes: (i) 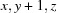; (ii) 
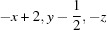
.

**Fig. 1.**
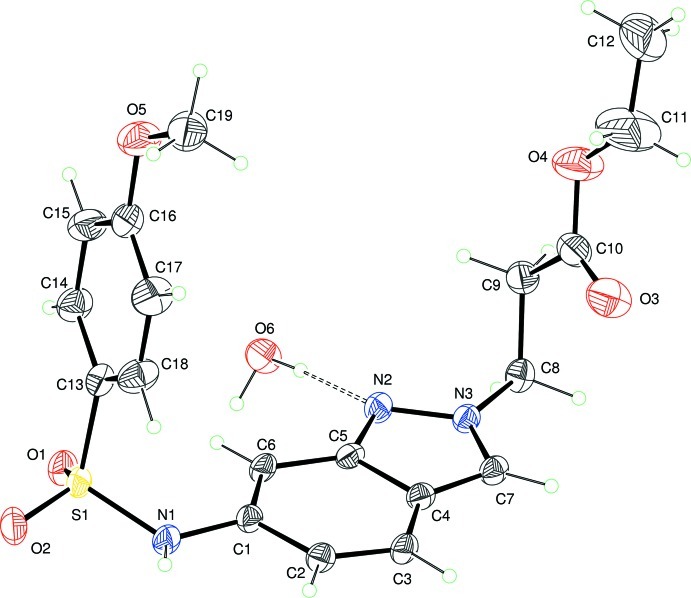
Molecular structure of the title compound. Displacement ellipsoids are drawn at the 50% probability level. H atoms are represented as small circles.

**Fig. 2.**
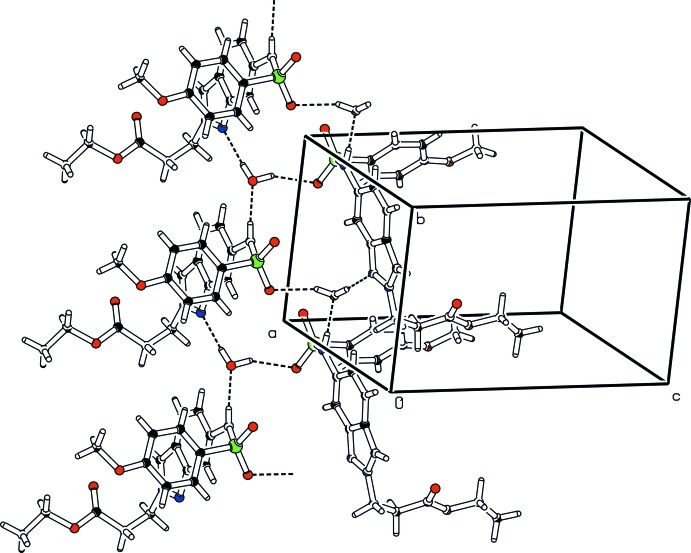
A view of crystal packing showing the water units linking the organic molecules in chains along the b axis.

## supplementary crystallographic information

### Comment

Sulfonamides constitute an important class of drugs. They possess various types of pharmacological activities such as antibacterial (Gadad *et al.*, 2000), anti-carbonic anhydrase (Brzozowski *et al.*, 2010), hypoglycemic (Drew, 2000), and anticancer activity (Garaj *et al.*, 2005). Recently, our research group has reported the synthesis of some new *N*-(6(4)-indazolyl)aylsulfonamide derivatives. Some of these compounds showed an important antiproliferative activity against some human and murine cell lines (Abbassi *et al.*, 2012; Bouissane *et al.*, 2006).

The crystal structure of the Ethyl-3-[6-(4-methoxybenzenesulfonamido)-2*H*- indazol-2-yl]-propanoate monohydrate is built up from two fused five- and six-membered rings (N2 N3 C1 to C7) virtually coplanar, with a maximum deviation of 0.021 (2) A Å for C2 atom as shown in Fig.1. Moreover, the two cycles are nearly perpendicular to the plan through the atoms forming the propionic acid ester group (O3O4 C9 to C11) and to benzene ring (C13 to C18) as indicated by the dihedral angles between them of 75.62 (8)° and 82.45 (8)° respectively. As a matter of fact, the molecule has a U shaped geometry.

The cohesion of the crystal structure is ensured by three classic strong hydrogen bonds between the water and the organic molecules: O6–H6A···N2, N1–H1···O6 and O6–H6B···O1, as shown in Fig.2 and Table 2.

### Experimental

A mixture of ethyl 3-(6-nitro-2*H*-indazol-2-yl)propanoate 1 (1.22 mmol) and anhydrous SnCl_2_ (1.1 g, 6.1 mmol) in 25 ml of absolute ethanol was heated at 333 K for 3 h. After reduction, the starting material disappeared, and the solution was allowed to cool down. The pH was made slightly basic (pH 7–8) by addition of 5% aqueous potassium bicarbonate before extraction with ethyl acetate. The organic phase was washed with brine and dried over magnesium sulfate. The solvent was removed to afford the amine, which was immediately dissolved in pyridine (5 ml) and then reacted with 4-methoxybenzenesulfonyl chloride (1.25 mmol) at room temperature for 24 h. After the reaction mixture was concentrated *in vacuo*, the resulting residue was purified by flash chromatography (eluted with Ethyl acetate: Hexane 1:9).

### Refinement

The structure is solved by direct method technique and refined by full-matrix least-squares using *SHELXS97* and *SHELXL97* program packages. H atoms were located in a difference map and treated as riding with C–H = 0.96 Å, C–H = 0.97 Å, C–H = 0.93 Å and N–H = 0.86 Å for methyl, methylene, aromatic CH and NH respectively. All hydrogen with *U*_iso_(H) = 1.2 *U*_eq_ (aromatic, methylene)and *U*_iso_(H) = 1.5 *U*_eq_ for methyl. The space group is not centro symmetric and the polar axis restraint is generated automatically by *SHELXL* program. The 1779 Friedel opposites reflections are not merged. The atomic displacement parameters of the C12 atom are quite large because it is a termial methyl that vibrates.

### Crystal data

**Table:**

C_19_H_21_N_3_O_5_S·H_2_O	*F*(000) = 444
*M**_r_* = 421.46	*D*_x_ = 1.369 Mg m^−^^3^
Monoclinic, *P*2_1_	Mo *K*α radiation, λ = 0.71073 Å
Hall symbol: p 2yb	Cell parameters from 4021 reflections
*a* = 9.0248 (3) Å	θ = 2.3–26.4°
*b* = 8.7602 (3) Å	µ = 0.20 mm^−^^1^
*c* = 13.1792 (4) Å	*T* = 296 K
β = 101.062 (2)°	Block, colourless
*V* = 1022.58 (6) Å^3^	0.42 × 0.37 × 0.28 mm
*Z* = 2	

### Data collection

**Table:**

Bruker X8 APEX diffractometer	3887 reflections with *I* > 2σ(*I*)
Radiation source: fine-focus sealed tube	*R*_int_ = 0.027
Graphite monochromator	θ_max_ = 26.4°, θ_min_ = 2.3°
φ and ω scans	*h* = −11→11
10050 measured reflections	*k* = −10→10
4021 independent reflections	*l* = −16→16

### Refinement

**Table:**

Refinement on *F*^2^	Secondary atom site location: difference Fourier map
Least-squares matrix: full	Hydrogen site location: inferred from neighbouring sites
*R*[*F*^2^ > 2σ(*F*^2^)] = 0.031	H-atom parameters constrained
*wR*(*F*^2^) = 0.077	*w* = 1/[σ^2^(*F*_o_^2^) + (0.037*P*)^2^ + 0.2529*P*] where *P* = (*F*_o_^2^ + 2*F*_c_^2^)/3
*S* = 1.05	(Δ/σ)_max_ < 0.001
4021 reflections	Δρ_max_ = 0.28 e Å^−^^3^
262 parameters	Δρ_min_ = −0.28 e Å^−^^3^
1 restraint	Absolute structure: Flack (1983), 1779 Friedel pairs
Primary atom site location: structure-invariant direct methods	Flack parameter: 0.03 (6)

### Special details

**Table:**

Geometry. All e.s.d.'s (except the e.s.d. in the dihedral angle between two l.s. planes) are estimated using the full covariance matrix. The cell e.s.d.'s are taken into account individually in the estimation of e.s.d.'s in distances, angles and torsion angles; correlations between e.s.d.'s in cell parameters are only used when they are defined by crystal symmetry. An approximate (isotropic) treatment of cell e.s.d.'s is used for estimating e.s.d.'s involving l.s. planes.
Refinement. Refinement of *F*^2^ against all reflections. The weighted *R*-factor *wR* and goodness of fit *S* are based on *F*^2^, conventional *R*-factors *R* are based on *F*, with *F* set to zero for negative *F*^2^. The threshold expression of *F*^2^ > 2σ(*F*^2^) is used only for calculating *R*-factors(gt) *etc*. and is not relevant to the choice of reflections for refinement. *R*-factors based on *F*^2^ are statistically about twice as large as those based on *F*, and *R*- factors based on all data will be even larger.

### Fractional atomic coordinates and isotropic or equivalent isotropic displacement parameters (Å^2^)

**Table:**

	*x*	*y*	*z*	*U*_iso_*/*U*_eq_	
C1	0.75199 (19)	0.7888 (2)	0.11333 (12)	0.0197 (4)	
C2	0.61152 (18)	0.8667 (2)	0.10899 (12)	0.0216 (3)	
H2	0.6095	0.9726	0.1039	0.026*	
C3	0.4816 (2)	0.7914 (2)	0.11201 (13)	0.0223 (4)	
H3	0.3909	0.8438	0.1071	0.027*	
C4	0.48733 (19)	0.6302 (2)	0.12290 (12)	0.0202 (3)	
C5	0.62772 (18)	0.5536 (2)	0.12955 (12)	0.0191 (3)	
C6	0.76182 (19)	0.6328 (2)	0.12325 (13)	0.0214 (4)	
H6	0.8528	0.5817	0.1257	0.026*	
C7	0.3845 (2)	0.5150 (2)	0.13056 (13)	0.0215 (4)	
H7	0.2815	0.5268	0.1283	0.026*	
C8	0.4016 (2)	0.2317 (2)	0.15290 (14)	0.0241 (4)	
H8A	0.4322	0.1643	0.1022	0.029*	
H8B	0.2921	0.2363	0.1386	0.029*	
C9	0.4548 (2)	0.1655 (2)	0.25982 (14)	0.0273 (4)	
H9A	0.4299	0.0577	0.2586	0.033*	
H9B	0.5638	0.1746	0.2782	0.033*	
C10	0.3852 (2)	0.2433 (2)	0.34120 (15)	0.0316 (4)	
C11	0.3662 (4)	0.2412 (4)	0.5169 (2)	0.0783 (11)	
H11A	0.4110	0.3401	0.5363	0.094*	
H11B	0.2581	0.2546	0.4950	0.094*	
C12	0.3972 (3)	0.1377 (5)	0.6044 (2)	0.0764 (10)	
H12A	0.3521	0.1767	0.6594	0.115*	
H12B	0.5043	0.1290	0.6277	0.115*	
H12C	0.3556	0.0391	0.5839	0.115*	
C13	1.07596 (19)	0.8265 (2)	0.27877 (13)	0.0236 (4)	
C14	1.1631 (2)	0.7069 (2)	0.32717 (15)	0.0309 (4)	
H14	1.2039	0.6351	0.2884	0.037*	
C15	1.1882 (2)	0.6960 (3)	0.43361 (15)	0.0355 (5)	
H15	1.2487	0.6179	0.4668	0.043*	
C16	1.1239 (2)	0.8006 (2)	0.49155 (15)	0.0304 (4)	
C17	1.0371 (3)	0.9201 (3)	0.44266 (16)	0.0367 (5)	
H17	0.9944	0.9908	0.4812	0.044*	
C18	1.0144 (2)	0.9330 (2)	0.33620 (16)	0.0342 (5)	
H18	0.9577	1.0136	0.3031	0.041*	
C19	1.0760 (3)	0.8738 (3)	0.65678 (15)	0.0430 (6)	
H19A	1.0906	0.8351	0.7261	0.065*	
H19B	0.9700	0.8764	0.6276	0.065*	
H19C	1.1167	0.9751	0.6576	0.065*	
N1	0.87396 (16)	0.88540 (16)	0.10220 (11)	0.0222 (3)	
H1	0.8609	0.9762	0.1044	0.027*	
N2	0.61166 (17)	0.40098 (17)	0.14153 (11)	0.0224 (3)	
N3	0.46167 (16)	0.38390 (16)	0.14178 (10)	0.0202 (3)	
O1	1.08832 (15)	0.70535 (16)	0.10224 (10)	0.0269 (3)	
O2	1.13045 (15)	0.98256 (17)	0.12154 (11)	0.0315 (3)	
O3	0.29906 (17)	0.3482 (2)	0.32659 (11)	0.0450 (4)	
O4	0.4297 (2)	0.1772 (2)	0.43247 (11)	0.0520 (5)	
O5	1.15167 (18)	0.77637 (19)	0.59538 (11)	0.0411 (4)	
O6	0.83323 (15)	0.19739 (15)	0.10176 (10)	0.0297 (3)	
H6A	0.7633	0.2565	0.1151	0.036*	
H6B	0.8488	0.2280	0.0427	0.036*	
S1	1.05094 (4)	0.84904 (5)	0.14406 (3)	0.02171 (10)	

### Atomic displacement parameters (Å^2^)

**Table:**

	*U*^11^	*U*^22^	*U*^33^	*U*^12^	*U*^13^	*U*^23^
C1	0.0167 (9)	0.0247 (8)	0.0181 (8)	0.0017 (7)	0.0040 (7)	−0.0004 (6)
C2	0.0208 (8)	0.0207 (8)	0.0237 (8)	0.0050 (7)	0.0055 (6)	0.0004 (7)
C3	0.0171 (9)	0.0250 (8)	0.0257 (9)	0.0078 (7)	0.0061 (7)	0.0006 (7)
C4	0.0192 (9)	0.0241 (9)	0.0173 (8)	0.0040 (7)	0.0038 (7)	0.0000 (6)
C5	0.0181 (8)	0.0219 (8)	0.0166 (8)	0.0047 (7)	0.0015 (6)	0.0002 (6)
C6	0.0157 (8)	0.0243 (9)	0.0243 (9)	0.0078 (7)	0.0044 (7)	0.0019 (7)
C7	0.0192 (9)	0.0253 (9)	0.0202 (8)	0.0051 (7)	0.0044 (7)	−0.0011 (7)
C8	0.0209 (9)	0.0225 (9)	0.0287 (9)	−0.0006 (7)	0.0043 (7)	−0.0027 (7)
C9	0.0273 (10)	0.0234 (9)	0.0319 (10)	0.0020 (7)	0.0073 (8)	0.0031 (7)
C10	0.0330 (11)	0.0330 (10)	0.0309 (10)	0.0010 (9)	0.0112 (8)	0.0053 (8)
C11	0.110 (3)	0.097 (2)	0.0363 (14)	0.036 (2)	0.0350 (16)	0.0102 (15)
C12	0.0642 (19)	0.129 (3)	0.0388 (14)	−0.015 (2)	0.0174 (13)	0.0096 (17)
C13	0.0187 (8)	0.0259 (9)	0.0256 (8)	−0.0004 (7)	0.0026 (7)	−0.0019 (7)
C14	0.0290 (11)	0.0319 (10)	0.0319 (10)	0.0091 (8)	0.0062 (8)	0.0010 (8)
C15	0.0344 (11)	0.0375 (11)	0.0334 (10)	0.0094 (9)	0.0033 (9)	0.0082 (9)
C16	0.0251 (10)	0.0354 (10)	0.0289 (10)	−0.0046 (7)	0.0010 (8)	0.0003 (7)
C17	0.0404 (12)	0.0377 (11)	0.0321 (10)	0.0088 (9)	0.0072 (9)	−0.0068 (8)
C18	0.0362 (12)	0.0331 (10)	0.0319 (10)	0.0124 (9)	0.0028 (9)	−0.0011 (8)
C19	0.0374 (12)	0.0635 (17)	0.0277 (10)	−0.0016 (11)	0.0051 (8)	−0.0046 (10)
N1	0.0180 (8)	0.0188 (7)	0.0298 (7)	0.0047 (5)	0.0047 (6)	0.0040 (6)
N2	0.0190 (8)	0.0228 (7)	0.0259 (7)	0.0033 (6)	0.0053 (6)	0.0013 (6)
N3	0.0178 (7)	0.0217 (8)	0.0209 (7)	0.0018 (6)	0.0033 (5)	0.0001 (5)
O1	0.0208 (7)	0.0314 (7)	0.0300 (7)	0.0056 (6)	0.0084 (5)	−0.0007 (6)
O2	0.0203 (7)	0.0324 (7)	0.0438 (8)	−0.0005 (6)	0.0114 (6)	0.0073 (6)
O3	0.0536 (9)	0.0433 (8)	0.0434 (8)	0.0182 (9)	0.0227 (7)	0.0073 (8)
O4	0.0646 (11)	0.0640 (11)	0.0303 (8)	0.0236 (9)	0.0165 (8)	0.0126 (7)
O5	0.0444 (9)	0.0506 (9)	0.0262 (8)	0.0047 (7)	0.0020 (6)	0.0013 (6)
O6	0.0334 (8)	0.0249 (7)	0.0335 (7)	0.0082 (6)	0.0133 (6)	0.0030 (5)
S1	0.0152 (2)	0.0246 (2)	0.0263 (2)	0.00303 (17)	0.00648 (15)	0.00187 (18)

### Geometric parameters (Å, º)

**Table:**

C1—C6	1.374 (2)	C12—H12A	0.9600
C1—N1	1.418 (2)	C12—H12B	0.9600
C1—C2	1.431 (2)	C12—H12C	0.9600
C2—C3	1.353 (3)	C13—C18	1.383 (2)
C2—H2	0.9300	C13—C14	1.390 (3)
C3—C4	1.419 (2)	C13—S1	1.7572 (17)
C3—H3	0.9300	C14—C15	1.381 (3)
C4—C7	1.387 (2)	C14—H14	0.9300
C4—C5	1.422 (2)	C15—C16	1.389 (3)
C5—N2	1.357 (2)	C15—H15	0.9300
C5—C6	1.411 (2)	C16—O5	1.360 (2)
C6—H6	0.9300	C16—C17	1.390 (3)
C7—N3	1.337 (2)	C17—C18	1.383 (3)
C7—H7	0.9300	C17—H17	0.9300
C8—N3	1.458 (2)	C18—H18	0.9300
C8—C9	1.514 (3)	C19—O5	1.436 (3)
C8—H8A	0.9700	C19—H19A	0.9600
C8—H8B	0.9700	C19—H19B	0.9600
C9—C10	1.506 (3)	C19—H19C	0.9600
C9—H9A	0.9700	N1—S1	1.6183 (14)
C9—H9B	0.9700	N1—H1	0.8050
C10—O3	1.196 (3)	N2—N3	1.363 (2)
C10—O4	1.326 (2)	O1—S1	1.4399 (14)
C11—C12	1.451 (4)	O2—S1	1.4327 (14)
C11—O4	1.458 (3)	O6—H6A	0.8601
C11—H11A	0.9700	O6—H6B	0.8600
C11—H11B	0.9700		
			
C6—C1—N1	124.49 (16)	H12A—C12—H12B	109.5
C6—C1—C2	121.23 (17)	C11—C12—H12C	109.5
N1—C1—C2	114.23 (15)	H12A—C12—H12C	109.5
C3—C2—C1	122.16 (17)	H12B—C12—H12C	109.5
C3—C2—H2	118.9	C18—C13—C14	120.58 (17)
C1—C2—H2	118.9	C18—C13—S1	119.33 (14)
C2—C3—C4	118.27 (17)	C14—C13—S1	120.01 (14)
C2—C3—H3	120.9	C15—C14—C13	119.13 (18)
C4—C3—H3	120.9	C15—C14—H14	120.4
C7—C4—C3	135.97 (17)	C13—C14—H14	120.4
C7—C4—C5	104.64 (15)	C14—C15—C16	120.55 (19)
C3—C4—C5	119.38 (16)	C14—C15—H15	119.7
N2—C5—C6	127.09 (15)	C16—C15—H15	119.7
N2—C5—C4	111.00 (15)	O5—C16—C15	115.76 (18)
C6—C5—C4	121.91 (16)	O5—C16—C17	124.24 (18)
C1—C6—C5	117.01 (16)	C15—C16—C17	120.01 (18)
C1—C6—H6	121.5	C18—C17—C16	119.51 (18)
C5—C6—H6	121.5	C18—C17—H17	120.2
N3—C7—C4	106.95 (15)	C16—C17—H17	120.2
N3—C7—H7	126.5	C17—C18—C13	120.20 (18)
C4—C7—H7	126.5	C17—C18—H18	119.9
N3—C8—C9	112.72 (15)	C13—C18—H18	119.9
N3—C8—H8A	109.0	O5—C19—H19A	109.5
C9—C8—H8A	109.0	O5—C19—H19B	109.5
N3—C8—H8B	109.0	H19A—C19—H19B	109.5
C9—C8—H8B	109.0	O5—C19—H19C	109.5
H8A—C8—H8B	107.8	H19A—C19—H19C	109.5
C10—C9—C8	112.84 (16)	H19B—C19—H19C	109.5
C10—C9—H9A	109.0	C1—N1—S1	125.60 (12)
C8—C9—H9A	109.0	C1—N1—H1	117.6
C10—C9—H9B	109.0	S1—N1—H1	108.9
C8—C9—H9B	109.0	C5—N2—N3	103.70 (13)
H9A—C9—H9B	107.8	C7—N3—N2	113.70 (14)
O3—C10—O4	123.80 (19)	C7—N3—C8	126.97 (15)
O3—C10—C9	125.49 (18)	N2—N3—C8	119.33 (14)
O4—C10—C9	110.70 (17)	C10—O4—C11	115.4 (2)
C12—C11—O4	108.8 (3)	C16—O5—C19	117.30 (17)
C12—C11—H11A	109.9	H6A—O6—H6B	104.5
O4—C11—H11A	109.9	O2—S1—O1	118.09 (7)
C12—C11—H11B	109.9	O2—S1—N1	105.63 (8)
O4—C11—H11B	109.9	O1—S1—N1	109.27 (8)
H11A—C11—H11B	108.3	O2—S1—C13	109.18 (9)
C11—C12—H12A	109.5	O1—S1—C13	107.13 (8)
C11—C12—H12B	109.5	N1—S1—C13	107.07 (8)
			
C6—C1—C2—C3	−1.5 (3)	C14—C13—C18—C17	−1.1 (3)
N1—C1—C2—C3	175.96 (15)	S1—C13—C18—C17	−177.88 (17)
C1—C2—C3—C4	1.9 (3)	C6—C1—N1—S1	−26.6 (2)
C2—C3—C4—C7	178.80 (18)	C2—C1—N1—S1	155.99 (12)
C2—C3—C4—C5	−0.4 (3)	C6—C5—N2—N3	−179.35 (15)
C7—C4—C5—N2	−0.54 (19)	C4—C5—N2—N3	0.25 (18)
C3—C4—C5—N2	178.89 (15)	C4—C7—N3—N2	−0.50 (19)
C7—C4—C5—C6	179.08 (15)	C4—C7—N3—C8	179.99 (15)
C3—C4—C5—C6	−1.5 (2)	C5—N2—N3—C7	0.16 (18)
N1—C1—C6—C5	−177.59 (14)	C5—N2—N3—C8	179.70 (14)
C2—C1—C6—C5	−0.4 (2)	C9—C8—N3—C7	−111.10 (19)
N2—C5—C6—C1	−178.61 (17)	C9—C8—N3—N2	69.41 (19)
C4—C5—C6—C1	1.8 (2)	O3—C10—O4—C11	0.0 (4)
C3—C4—C7—N3	−178.7 (2)	C9—C10—O4—C11	−178.6 (2)
C5—C4—C7—N3	0.61 (18)	C12—C11—O4—C10	167.3 (3)
N3—C8—C9—C10	70.9 (2)	C15—C16—O5—C19	−174.48 (19)
C8—C9—C10—O3	−1.3 (3)	C17—C16—O5—C19	5.2 (3)
C8—C9—C10—O4	177.17 (17)	C1—N1—S1—O2	−176.12 (14)
C18—C13—C14—C15	−0.3 (3)	C1—N1—S1—O1	55.87 (16)
S1—C13—C14—C15	176.48 (16)	C1—N1—S1—C13	−59.84 (16)
C13—C14—C15—C16	1.8 (3)	C18—C13—S1—O2	66.47 (17)
C14—C15—C16—O5	177.83 (19)	C14—C13—S1—O2	−110.37 (16)
C14—C15—C16—C17	−1.9 (3)	C18—C13—S1—O1	−164.56 (15)
O5—C16—C17—C18	−179.2 (2)	C14—C13—S1—O1	18.60 (18)
C15—C16—C17—C18	0.5 (3)	C18—C13—S1—N1	−47.44 (18)
C16—C17—C18—C13	1.0 (3)	C14—C13—S1—N1	135.72 (15)

### Hydrogen-bond geometry (Å, º)

**Table:**

*D*—H···*A*	*D*—H	H···*A*	*D*···*A*	*D*—H···*A*
O6—H6*A*···N2	0.86	1.94	2.8029 (19)	176
N1—H1···O6^i^	0.81	1.95	2.7575 (19)	177
O6—H6*B*···O1^ii^	0.86	2.10	2.9094 (17)	156

Symmetry codes: (i) *x*, *y*+1, *z*; (ii) −*x*+2, *y*−1/2, −*z*.
